# Whole-Exome Sequencing Identifies Candidate Genomic Features Associated with Response to Platinum-Based Chemotherapy and Ixabepilone-Based Treatment in Ovarian Cancer

**DOI:** 10.3390/ijms27177524

**Published:** 2026-08-22

**Authors:** Tobias M. P. Hartwich, Stefania Bellone, Orazio De Tommasi, Sarah Ottum, Victoria Ettorre, Michelle Greenman, Namrata Sethi, Cem Demirkiran, Kevin Y. Yang, Ammal Abbasi, Ludmil B. Alexandrov, Alessandro D. Santin

**Affiliations:** 1 Department of Obstetrics, Gynecology and Reproductive Sciences, Yale University School of Medicine, New Haven, CT 06520, USA; tobias.hartwich@yale.edu (T.M.P.H.); namrata.sethi@yale.edu (N.S.); kevin.yang@yale.edu (K.Y.Y.); 2 Clinic of Gynecology and Obstetrics, Department of Women and Children’s Health, University of Padua, 35100 Padua, Italy; 3 Department of Cellular and Molecular Medicine, University of California San Diego, La Jolla, CA 92093, USA

**Keywords:** ovarian cancer, whole exome sequencing, next-generation sequencing, chemo-response, chemo-resistance

## Abstract

Carboplatin/paclitaxel (CP) chemotherapy is the cornerstone of therapy for advanced stage ovarian cancer (OC). However, despite initial sensitivity, this regimen cannot avoid the emergence of resistance. Ixabepilone ± bevacizumab (IB) is a combination recently added to NCCN guidelines for the treatment of platinum-resistant OC. It would be desirable to identify biomarkers able to differentiate patients who are resistant to CP and IB, and biomarkers that identify which patients may benefit from IB treatment. We analyzed whole-exome-sequencing (WES) data from 49 OC patients exposed to CP, including 28 platinum-sensitive vs. 21 platinum-resistant, and 31 additional platinum-resistant patients, including 16 responders (i.e., CR/PR) vs. 15 non-responders (SD/PD) to ixabepilone ± bevacizumab. Comprehensive genetic analyses were performed to identify alterations correlated with resistance to CP and IB. WES analysis of CP responders vs. non-responders revealed differences in HRD-signatures (*p* < 0.05), OS (*p* < 0.005) and gain/loss-of-function in multiple genes associated with tumor growth/progression including but not limited to *ACVR2A*, *INHBA*, *MAP3K7*, *ATG5*, *SGK1*, *FYN*, *RSPO3*, *NOD1* and *LRRK2*. WES analysis of platinum-resistant IB-treated patients revealed additional nominally significant genes and deranged pathways including gains in the *DROSHA* and *SDHA* genes in responders vs. non-responders (*p* < 0.05). Patients harboring HRD-signatures showed significantly higher sensitivity to CP and prolonged survival compared to HRD-negative patients. Alterations in genes associated with tumor growth/progression correlated with resistance to CP regimen and may represent novel “druggable” candidate biomarkers for the targeted treatment of CP/IB-resistant patients. Further validation in independent cohorts and preclinical experiments in CP/IB-resistant models are warranted to establish the clinical utility of these findings.

## 1. Introduction

Ovarian cancer remains one of the deadliest gynecologic cancers in the United States of America with 21,010 new cases in 2026 and 12,450 estimated deaths [1]. Although the majority of ovarian cancer patients initially respond to the first-line gold standard chemotherapy treatment based on the combination of carboplatin and paclitaxel, due to late-stage initial diagnosis, survival rates remain disappointing with 70% of patients developing recurrence within 2 years [2].

Treatment strategy for recurrent ovarian cancer is based on platinum sensitivity which is defined in the clinic by the duration of progression-free survival (PFS) after the completion of the platinum-based regimen. Patients with a PFS less than 6 months are considered to harbor a platinum-resistant tumor, while PFS more than 6 months reveals platinum sensitivity [3]. While 70 to 80% of chemotherapy-naïve ovarian cancer patients respond initially to the combination of carboplatin and paclitaxel, there are currently no effective methods to identify platinum resistance in the upfront setting or resistance to additional lines of chemotherapy in the recurrent setting [4].

High-grade serous ovarian cancer (HGSOC) is the most common histological type of ovarian tumor. This variant is characterized by high genomic instability with a high frequency of gain/loss genomic copy number variations (CNVs) and loss of heterozygosity (LOH). *TP53* mutations are reported in over 90% of cases, while germline and somatic *BRCA1* or *BRCA2* mutations are detected in 15 to 25% of ovarian tumors [5]. In comprehensive next-generation sequencing studies (i.e., WES) evaluating mutations in known ovarian cancer driver genes, 77% were found to be transmitted from primary tumors to metastatic tumors, and 80% from primary to recurrent tumors, indicating that driver mutations are commonly retained during ovarian cancer evolution [6]. Moreover, chemotherapy-resistant ovarian tumors demonstrated higher prevalence of *MYC* and *PIK3CA* amplifications, and these changes persisted during progression to metastasis and were even more significant in recurrent tumors [6]. However, despite increasing knowledge of ovarian cancer molecular features, information about prognostic or predictive genetic signatures of resistance to the initial platinum treatment or to subsequent lines of chemotherapy, which could be used for personalizing targeted treatment modalities, remain limited.

In an attempt to fill this gap in knowledge, the present study analyzed the whole-exome gene mutation spectra in different cohorts of surgically resected ovarian cancer patients including mostly chemotherapy-naïve HGSOC treated with carboplatin and paclitaxel, as well as a cohort of platinum-resistant/refractory ovarian cancer patients treated in the recurrent setting with the combination of ixabepilone ± bevacizumab, a combination regimen recently added to NCCN guidelines for the treatment of platinum-resistant ovarian cancer patients [7]. We compared genetic alterations in individual genes and pathways, copy number variations (CNVs), and mutational signatures in responder vs. non-responder patients, providing candidate leads for follow-up validation studies focused on utilizing somatic genetic profiles for the precision oncology treatment of high-grade ovarian cancer patients.

## 2. Results

### 2.1. Patients’ Characteristics

The main clinical characteristics of the ovarian carcinoma patients included in our analysis (i.e., cohort 1) and the platinum-resistant/refractory (i.e., cohort 2) are described in Table 1 and Table 2, respectively. Cohort 1 included 49 ovarian cancer patients exposed to CP, including 28 platinum-sensitive (i.e., CR/PR with PFI ≥ 6 mo) and 21 platinum-resistant (PD with PFI < 6 mo) patients. Cohort 2 included a total of 31 platinum-resistant/refractory patients, 16 responding with a CR/PR to the ixabepilone ± bevacizumab combination vs. 15 non-responding (SD/PD) patients. The median age of patients at diagnosis for cohort 1 was 58 years (range 24–80) while for cohort 2 it was 65 years (range 39–76); about 94.3% of patients at diagnosis had advanced disease with FIGO stage III or IV in cohort 1 vs. 96.8% in cohort 2 and most of the patients (92.5% and 87.1%, respectively) harbored a HGSOC histologic subtype and were grade 3. Caucasian/white represented 100% of cohort 1 patients vs. 74.2% in cohort 2.

### 2.2. Single Nucleotide Variant (SNV) Analysis

**Figure 1:**
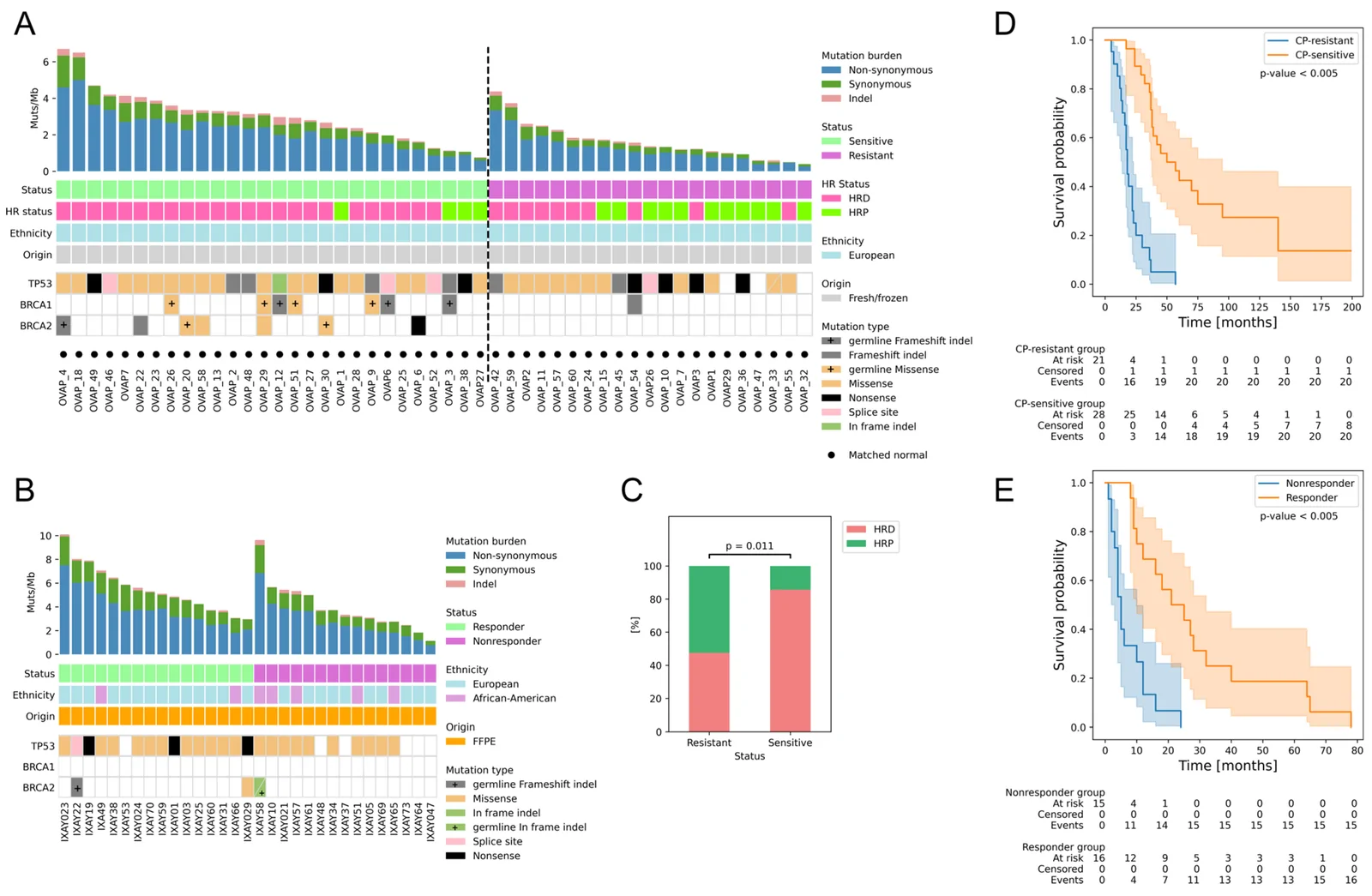
(**A**) Mutational landscape plot showing relevant mutations in cohort 1 including HR status obtained using ovaHRDscar. (**B**) Mutational landscape plot showing relevant mutations in cohort 2. (**C**) Distribution of HR status in CP-sensitive and CP-resistant groups. (**D**) Kaplan–Meier survival plot of cohort 1. (**E**) Kaplan–Meier survival plot of cohort 2.

Whole exome sequencing analysis was performed for the set of 49 primary ovarian cancer tumor samples with matched normal (cohort 1) as well as the set of 31 unmatched platinum-resistant/refractory ovarian cancer samples (cohort 2). In the first set of 49 samples (Figure 1A), sequenced from fresh/frozen tumor samples, 28 of the samples were classified as chemo-sensitive versus 21 chemo-resistant. No significant SNVs were found besides the *TP53* gene which was altered in 46/49 ovarian cancer samples (94%). The three samples without *TP53* alteration were part of the chemo-resistant cohort leading to a *TP53* alteration rate in this cohort of 18/21 (86%). The *BRCA1* gene presented SNVs in 1/49 samples (2%) as well as germline alterations in an additional 7/49 samples (14%). *BRCA2* presented SNVs in 4/49 samples (8%) as well as germline alterations in an additional 3/49 samples (6%). Overall, *BRCA1/2* alterations affected a total of 14/49 samples (29%) with almost all these mutations (93%) affecting samples in the chemo-sensitive cohort (13/14) (Figure 1A). In cohort 1 the overall tumor mutational burden in the chemo-sensitive group was slightly higher with an average of 2.4 +/− 0.2 mutations per megabase compared to 1.3 +/− 0.2 mutations per megabase in the chemo-resistant group, (mean +/− SEM, *p* < 0.001).

The second cohort of 31 samples (Figure 1B), sequenced from FFPE tumor samples, was derived from platinum-resistant/refractory patients enrolled within a clinical trial comparing the clinical activity of ixabepilone ± bevacizumab [8,9]. In total, 16 samples were classified as responsive to treatment with ixabepilone ± bevacizumab (i.e., CR/PR) versus 15 samples as non-responsive (i.e., SD/PD) using RECIST 1.1 criteria. *TP53* SNVs were present in 25/31 samples (81%) with 15/16 samples (94%) in the responsive cohort and 10/15 samples (67%) in the non-responsive cohort. There were very few *BRCA1/2* alterations in this sample set of platinum-resistant patients with none of the samples presenting with *BRCA1* alterations and only 3/31 samples (10%) presenting *BRCA2* alterations. This is in line with our findings in the CP-resistant group in cohort 1. In cohort 2, tumor mutational burden was not significantly different with a burden of 4.0 +/− 0.4 mutations per megabase and 2.9 +/− 0.4 (mean +/− SEM) in the responsive versus non-responsive cohorts.

### 2.3. Genetic Signatures of HRD in Ovarian Cancer Patients

Homologous recombination status in cohort 1 was assessed using the software ovaHRDscar (v0.1.0) with 34/49 samples (69%) categorized as homologous recombination deficient (HRD) and the remaining 15/49 samples (31%) as proficient. The HRD calls were additionally validated via a combination of de novo mutational signature extraction and HRProfiler, which uses HRD-specific mutation and copy number features. Overall concordance for HRD calls between the methods was very high at 97.4%. When we compared HR status between the chemo-sensitive and chemo-resistant cohorts using the chi-square test we found a statistically significant higher percentage of HR-deficient samples in the chemo-sensitive cohort (i.e., 24/28 samples = 86%) vs. only 10/21 samples in the CP-resistant subgroup (48%) (*p* = 0.011, Figure 1C).

### 2.4. Overall Survival and Copy Number Variant (CNV) Analysis of Cohort 1

**Figure 2:**
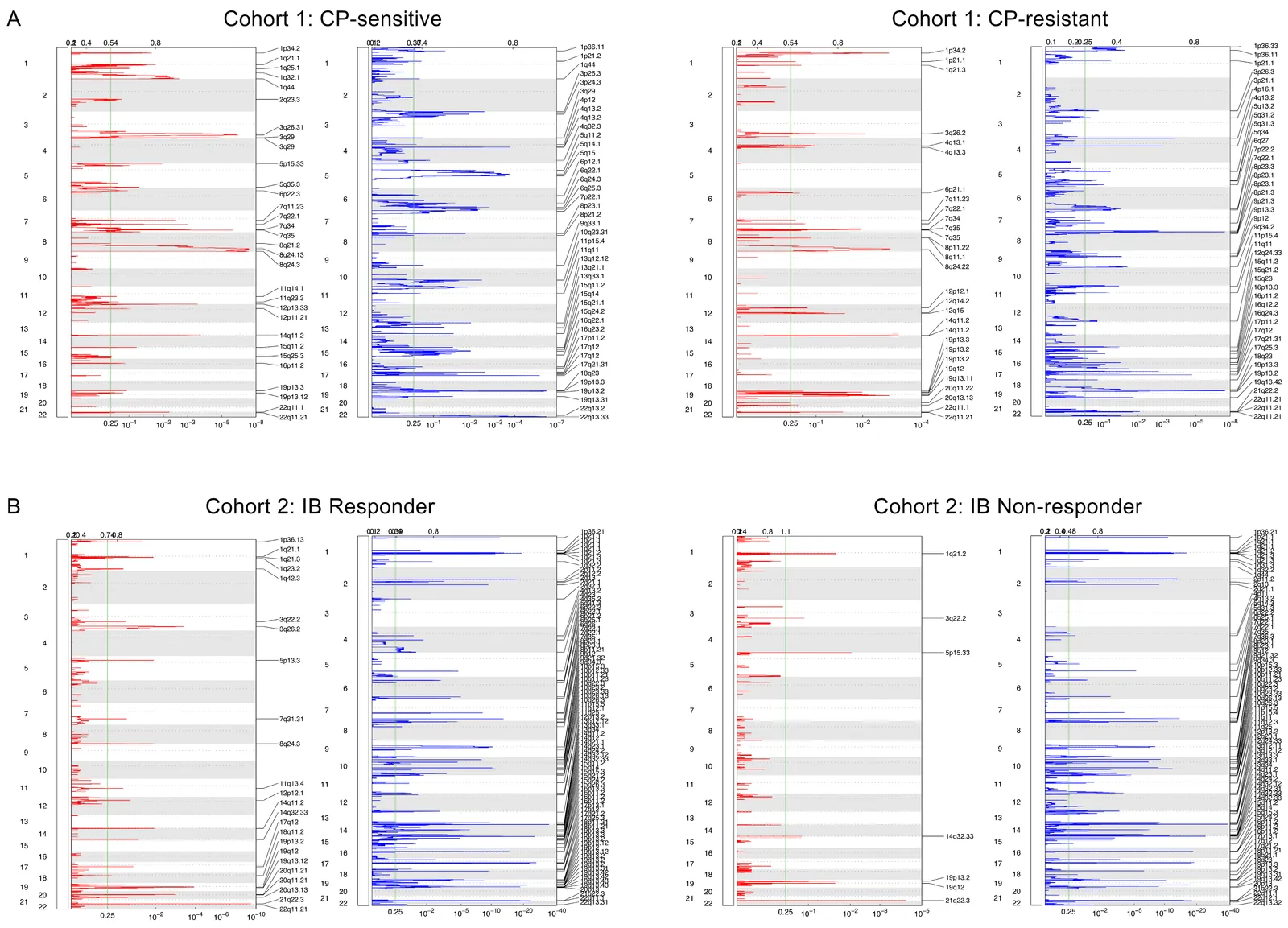
GISTIC plots of amplifications (red) and deletions (blue) in (**A**) cohort 1 in the CP-sensitive group (left) and the CP-resistant group (right), and (**B**) cohort 2 in the treatment-responsive group (left) and the non-responsive group (right).

Survival outcomes analysis of cohort 1 patients demonstrated, as expected, that platinum-sensitive women had significantly longer overall survival (OS) than platinum-resistant patients, with a median OS of 50 months in platinum-sensitive patients versus 18 months in platinum-resistant patients (log-rank *p* < 0.005, Figure 1D) with a hazard ratio for the platinum-resistant group of 12.0 (95% CI 4.9–29.5, *p* < 0.005). When we evaluated copy number variation (CNV) analysis in 49 primary ovarian cancer tumor samples with matched normal, with 21 tumors classified as chemo-resistant and 28 tumors as chemo-sensitive, we found a large number of amplifications and deletions across the whole genome as analyzed by Genomic Identification of Significant Targets in Cancer (GISTIC). In particular, the cytogenic bands 3q26, 8q23–24 and 14q11 among the amplification peaks as well as 1p36, 4p16, 7p22, 8p21–23, 9q34, 11p15 and 19p13 among the deletion peaks were identified as the ones with the highest significance. Both the chemo-sensitive cohort as well as the chemo-resistant cohort show peaks in these cytogenetic bands as seen in Figure 2A, with only two notable exceptions. The chemo-sensitive cohort did not show deletion peaks for the bands 9q34 (containing *NOTCH1*) and 4p16 (containing *FGFR3*) but instead showed a significant amplification peak for 4p16. Other notable differences were strong amplification peaks in 1q44, 3q29 and 11q23 (containing the oncogenes *ITPKB*, *AKT3*, *SMYD3*, *PGBD5*, *ETS1*, *FLI1*, *PAFAH1B2*, *ARHGEF12* and *BCL9L*) present in the chemo-sensitive cohort and in 19p13 and 19q12–13 (containing oncogenes *CALR*, *CCNE1*) in the chemo-resistant cohort. For deletions, the peaks in bands 5q11 and 5q14–15 (containing the tumor suppressors *APC*, *MAP3K1*, *MSH3*, *PIK3R1*, *RAD17*, *RASA1* and *ELL2*) stood out in the chemo-sensitive cohort with no peaks particularly prominent for only the chemo-resistant cohort.

**Figure 3:**
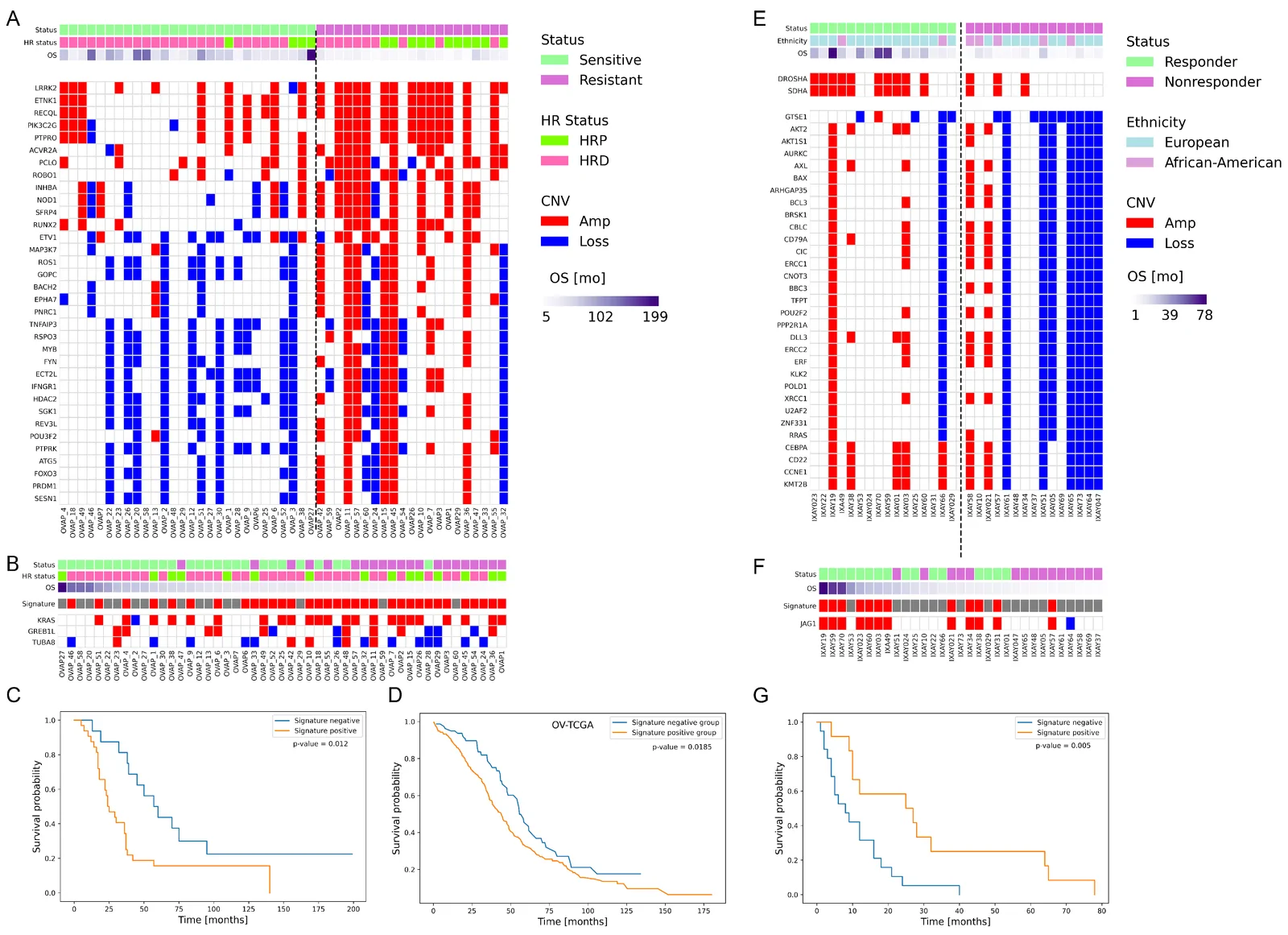
(**A**) Overview plot of gene level CNV in cohort 1 for cancer-related genes showing significant amplification in the CP-resistant group versus the CP-sensitive group. (**B**) Genetic signature associated with worse overall survival in cohort 1. Samples were determined to be signature positive if they exhibited at least one of the three features: *KRAS* amplification, *GREB1L* loss and/or *TUBA8* loss. (**C**) Kaplan–Meier survival curves for signature positive and negative patients in cohort 1 and (**D**) in the OV-TCGA data. (**E**) Overview plot of gene level CNV in cohort 2 for cancer-related genes statistically different between treatment-responsive and non-responsive groups. (**F**) Genetic signature associated with better overall survival in cohort 2. Samples were determined as signature positive if they exhibited *JAG1* amplification. (**G**) Kaplan–Meier survival plot for signature positive and negative patients in cohort 2.

Next, we looked at CNV events at the gene and sample level for known cancer-related genes as listed in OncoKB using chi-square tests to assess genes’ amplifications or deletions between the chemo-sensitive and chemo-resistant groups. We found 34 cancer-related genes nominally significantly (*p* < 0.05) amplified in the CP-resistant group, including but not limited to *ACVR2A*, *INHBA*, *MAP3K7*, *ATG5*, *SGK1*, *FYN*, *RSPO3*, *NOD1* and *LRRK2* (Figure 3A). However, none of the genes were considered significant after correction for multiple testing. Several of the genes identified as amplified in CP-resistant patients can be grouped into similar overarching pathways (Table 3). Analysis of copy number variation patterns and overall survival revealed an exploratory gene signature that correlates to poor prognosis in cohort 1 (Figure 3B). The signature consists of amplification in *KRAS*, deletion in *GREB1L* and deletion in *TUBA8* with patients considered signature positive if at least one of those conditions were met. Median OS was 57 months in the signature negative group versus 25 months in the signature positive group (*p* = 0.012, Figure 3C) with a hazard ratio for the signature positive group of 2.6 (95% CI 1.3–5.4, *p* = 0.01). When this signature was validated in a separate sample set from TCGA (i.e., OV-TCGA) we obtained similar results with median OS of 56 months for the signature negative group and of 44 months for the signature positive group (*p* = 0.0185, Figure 3D).

### 2.5. Overall Survival and Copy Number Variant (CNV) Analysis of Cohort 2

Overall survival (OS) in cohort 2 patients was, as expected, markedly prolonged in the IB responder group compared with non-responder group, with a median OS of 21 months versus 5 months, respectively (Figure 1E, log-rank *p* < 0.005), with a hazard ratio for the non-responder group of 4.7 (95% CI 1.7–13.1, *p* < 0.005). We next analyzed and compared WES data for CNV between the group of platinum-resistant patients responsive vs. non-responsive to IB. As expected, GISTIC gain/loss analysis results obtained from IB-treated patients demonstrated CNV genetic features similar to those identified in the CP-resistant group of cohort 1. Consistent with this view, amplification peaks in 19p13 and 19q12 were clearly recognizable as well as the absence of broad deletion peaks around 5q11/14–15 (Figure 2B). This similarity was also evident at the gene/sample level when looking at CNV distribution in genes that differentiated the CP-sensitive from the CP-resistant groups. Importantly, the gene level comparison between the IB responsive and non-responsive groups further reveals a group of genes that, although only nominally significant, in the non-responder group exhibits deletions in about half of the group, 7/15 samples (47%), versus only 1/16 samples (6%) in the responsive group (*p* < 0.05, Figure 3E). Two additional genes, *DROSHA* and *SDHA*, were found nominally significantly (*p* < 0.05) amplified in the IB-responsive versus the non-responsive group with 10/16 samples (63%) vs. 3/15 samples (20%), respectively, potentially demonstrating possible gain of function. When comparing CNVs against overall survival in cohort 2, we identified several genes correlated with better overall survival (Table 4). Among them were, for example, drivers of tumor progression and therapeutic resistance (*JAG1*, *MUC1*, *PARP1*), microtubule development (*CAMSAP2*, *CENPA*, *TBCE*), and actin cytoskeleton/structural integrity-related (*ACTA1*, *LMNA*, *SPTA1*). These genes allowed us to create an exploratory gene signature where gene amplification serves as a favorable biomarker for treatment response, correlating with a longer overall survival (OS) in cohort 2 patients. Figure 3F,G show a preliminary signature based on amplification in *JAG1*, which resulted in median OS of 25 months for the group with amplifications versus 8 months for the group without (*p* = 0.005) with a hazard ratio for the signature negative group of 2.4 (95% CI 1.0–5.9, *p* = 0.06), although additional studies are required to validate these results in larger cohorts.

## 3. Discussion

This study aimed to identify genetic characteristics to act as potential biomarkers for stratifying upfront platinum-responsive vs. -resistant ovarian cancer patients treated with CP as well as platinum refractory/resistant ovarian cancer patients treated with IB at the time of recurrence. Accordingly, we used WES to compare the genomic landscape between platinum responsive and resistant patients, encompassing single nucleotide variation, copy number variation, TMB and HRD genetic signatures. Furthermore, the impact of genomic alterations on the response to ixabepilone ± bevacizumab was also evaluated comparing responders vs. non-responders in platinum-refractory/resistant ovarian cancer patients.

Our data analysis shows that patients resistant to the CP combination have a significantly shorter overall survival when compared to CP-responsive patients and a small but significantly lower load of somatic mutations than CP-sensitive patients. Similarly, patients resistant to the IB combination demonstrated significantly shorter overall survival when compared to the IB responder subgroup (i.e., cohort 2). In both cohorts, alterations in *TP53* genes were detected in the overwhelming majority of ovarian cancer patients while *BRCA1* and *BRCA2* mutations were identified in 29% of the patients of cohort 1. BRCA-mutated ovarian cancer patients are notoriously sensitive to platinum-based chemotherapy (CP) because *BRCA1/2* mutations impair the homologous recombination repair pathway, making tumor cells dependent on less efficient DNA repair methods. Consistent with this view we found patients with tumors harboring *BRCA1/2* alterations to be significantly more represented in the CP-sensitive subgroup of cohort 1. Taken together, our SNV results align with the established literature regarding the molecular landscape of High-Grade Serous Ovarian Carcinoma [5,6] and support a prognostic and therapeutic role of *BRCA1/2* positivity of ovarian cancer patients treated with carboplatin and paclitaxel.

Mutational signatures extracted from WES data have recently demonstrated to provide major insights into the biological processes shaping the tumor genome, and can potentially inform patients’ sensitivity or resistance to targeted treatments in multiple tumor types [10]. Consistent with this view, our data analysis of cohort 1 patients demonstrated that women resistant to the platinum and taxane combination also have a significantly lower HRD when compared to platinum-sensitive patients. In our study we confirmed the pivotal role of mutations in homologous recombination repair genes in platinum sensitivity/resistance using multiple platforms. We used computational analysis of genomic scarring similar to Myriad testing, validated by analysis of mutational patterns, including single Base Substitution Signature 3 (SBS3) and WES-based HRD markers, as validated, reliable molecular markers for HRD [10]. Using this approach 34/49 (69%) samples were called HRD. These HRD results are similar to the approximately 50% score described in the TCGA dataset [5] as well as the 55% HRD score described in other large series of ovarian cancer patients [6]. Importantly, when we compared HRD status between the chemo-sensitive and chemo-resistant patients in cohorts 1 using the chi-square test we found a statistically significant higher percentage of HR-deficient samples in the chemo-sensitive vs. CP-resistant subgroup (*p* = 0.011). The higher percentage of HRD patients in the CP-sensitive subgroup is consistent with previous reports, demonstrating that HRD may not only predict response to PARPi [11] but may also represent a marker of sensitivity of platinum-based chemotherapy [12].

High-grade ovarian carcinoma is known to harbor a genome dominated by CNVs rather than SNVs. Consistent with this view, we found ovarian cancer genomes of both cohorts 1 and 2 to be characterized by profound chromosomal instability leading to widespread amplifications and deletions. Importantly, when we compared CNV differences between the CP-sensitive vs. CP-resistant subgroups we found 110 cancer-related genes nominally significantly amplified or deleted (*p* < 0.05). We identified copy number gains in multiple genes such as *ACVR2A*, *INHBA*, *MAP3K7*, *ATG5*, *SGK1*, *FYN*, *RSPO3*, *NOD1* and *LRRK2*, known to play key roles in cancer progression and signaling pathways associated with biologic aggressiveness and resistance to cytotoxic agents in multiple human tumors. Of interest, we found many of the functions of these cancer genes to be interconnected, broad biological processes, primarily focused on intracellular trafficking, vesicle dynamics, cell development, chronic inflammation, signaling cascades and resistance to treatment. For example, amplification/overexpression of Activin A receptor type 2A (*ACVR2A*), a key component of the Transforming Growth Factor-beta (*TGF-beta*) signaling pathway, has previously been linked to poor prognosis in ovarian cancer patients and identified as a potential biomarker for chemotherapy resistance [13]. Supporting the importance of this pathway, we found additional genes of the TGF-beta superfamily including Inhibin Subunit Beta A (*INHBA*), Mitogen-Activated Protein Kinase Kinase Kinase 7 (*MAP3K7*) and Autophagy-Related 5 (*ATG5*), to be nominally significantly amplified in CP-resistant tumors. The *INHBA* gene product is a known ligand that binds to the ACVR2A to regulate reproduction, development, cell proliferation, and wound healing and is associated with poor prognosis and disease progression in multiple cancers, including ovarian cancer [13]. It acts as a signaling molecule in the TGF-beta pathway, prompting epithelial–mesenchymal transition (EMT) and cancer cell migration and, accordingly, is a potent driver of malignant behavior in cancer cells [14]. Both MAP3K7, a kinase involved in TGF-beta-induced signal transduction, and ATG5, a core autophagy protein involved in autophagic vessel formation, have previously been independently linked to chemo-resistance in various cancers, including ovarian cancer [15,16].

We also found nominally significantly amplified genes that intersect or are downstream effectors of the PI3K/AKT pathway, namely Serum/Glucocorticoid Regulated Kinase 1 (*SGK1*), FYN Proto-Oncogene, Src Family Tyrosine Kinase (*FYN*) and R-Spondin 3 (*RSPO3*). *SGK1* amplification may represent a key mechanism driving tumor resistance to chemotherapy in cancers like platinum-resistant ovarian cancer (PROC). Indeed, by acting as a central node in glucocorticoid receptor (GR) signaling, SGK1 promotes cell survival and inhibits apoptosis, potentially explaining why patients with high GR activity derive significant clinical benefit from treatment with relacorilant. RSPO3 is a secreted protein that functions as a key modulator of the canonical Wnt/β-catenin signaling pathway. In the context of ovarian cancer, it has been linked to EMT, metastasis and generally worse outcomes via the PI3K/AKT pathway. FYN is a non-receptor tyrosine kinase with broad functional variety and, among others, plays a role in microtubule assembly, MEK/ERK signaling and apoptosis. Elevated expression of FYN has also been associated with chemo-resistance in ovarian cancer [17,18,19].

Another nominally significantly amplified set of genes identified in our CNV analysis is related to NF-κB/inflammatory signaling, including *LRRK2* and *NOD1*. High expression levels of the leucine-rich repeat kinase 2 (*LRRK2*) gene, a multidomain protein containing GTP-binding regulatory domain (ROC-COR) and a kinase domain, have been previously correlated with worse clinical outcomes in ovarian cancer and increased resistance to chemotherapy. Consistent with this view, reduced LRRK2 expression has been shown to inhibit cell progression and invasion of cancer cells and sensitize ovarian cancer cells to olaparib (a PARP inhibitor), suggesting that limiting LRRK2 activity pharmacologically could improve treatment outcomes [20]. Of interest, *NOD1* was nominally significantly amplified in the subgroup of CP-resistant ovarian tumors with its downstream signaling mediator *RIPK2* also strongly amplified in the same samples. It is worth noting that elevated expression of the nucleotide-binding oligomerization domain receptor 1 (NOD1) and RIPK2 have been previously reported to be associated with poor prognosis, increased tumor growth, and metastasis in ovarian cancer patients [21]. NOD1 promotes ovarian cancer progression by increasing cell proliferation, invasion, and migration. This occurs through the activation of the nuclear factor kappa B-light-chain-enhancer of activated B cells (NF-kappa B) and mitogen-activated protein kinase (MAPK) pathways and inducing production of proinflammatory cytokines like TNF-α and IL-6 [22]. Importantly, because NOD1 promotes tumor growth and metastasis and it has been associated with resistance to paclitaxel [23], it may represent a potential therapeutic target in CP-resistant patients. Consistent with this view, inhibition of the NOD1/RIPK2 signaling pathway has been suggested as a method to reduce tumor progression and improve survival outcomes in cancer patients [24].

We also found the *CCNE1* gene to be significantly amplified in the subset of CP-resistant tumors. Cyclin E1 overexpression is known to accelerate G1/S progression, thereby generating replication stress and DNA damage. Previous studies in ovarian cancer patients demonstrated that increased expression levels of cyclin E1 are associated with worse survival [25,26]. At the genomic level, previous studies have described high frequency of *CCNE1* gains in patients with primary platinum-resistant disease [27]. Importantly, multiple clinical trials are currently targeting *CCNE1*-amplified ovarian cancers using novel agents that exploit high replication stress, such as CDK2 inhibitors (e.g., BLU-222, PF-07104091) and combinations like PKMYT1 (lunresertib) with ATR inhibitors.

Of interest, several of the nominally significantly amplified genes in the CP-resistant group in cohort 1 are related to MEK/ERK signaling, such as *ETV1*, *EPHA7*, *FYN*, *MAP3K7* and *RUNX2* [28,29,30,31]. We also observed *KRAS* amplification rates of 62% (13/21) in the CP-resistant group and 36% (10/28) in the CP-sensitive group. When we created a gene signature combining *KRAS* amplification with loss of *GREB1L*, an estrogen-regulated gene for which upregulation is linked to improved clinical outcome in breast cancer [32], or loss of *TUBA8*, an isoform of α-tubulin, we were able to show improved survival in response to CP treatment in both our data and the OV-TCGA dataset. While there is a need for further validation, these data, taken together, suggest that an active MEK/ERK pathway may be related to CP resistance, paving the way for the future discovery and validation of new therapeutic targets for CP-resistant ovarian cancer patients.

We also observed that *DROSHA* (a key enzyme in microRNA processing) and *SDHA* (a subunit of the Succinate Dehydrogenase complex II) were nominally significantly amplified in IB-responsive patients compared to non-responsive ones. In this regard, *DROSHA* is typically associated with miRNA biogenesis, and involved in regulating tumor growth, responding to DNA damage, or acting as an oncogene in specific contexts, where its overexpression/amplification correlates with improved responsiveness to chemotherapy combinations [33,34]. In contrast, *SDHA* functions as a tumor suppressor, but its amplification may affect the metabolic profile of the tumor (Krebs cycle/oxidative phosphorylation) as observed in other cancer types, potentially sensitizing it to the combination of microtubule stabilization (ixabepilone) and anti-angiogenesis (bevacizumab) [35,36]. Taken together, these findings suggest that genomic profiling for *DROSHA* and *SDHA* amplification may have the potential to help identify patients who will benefit from the ixabepilone ± bevacizumab combination, particularly when other markers like beta III-tubulin (TUBB3) fail to predict response [8,9].

Despite the very interesting findings presented in our manuscript, it is important to acknowledge that there were several factors that imposed limitations on our ability to make broadly generalized statements based on our results. First of all, due to the exploratory nature of our study, the cohort sample sizes that were available for analysis were limited, with only 49 and 31 samples for cohorts 1 and 2 respectively. While both cohorts were mostly extracted from tissues collected of the primary tumor, several samples in each cohort had been exposed to 2–6 cycles of prior chemotherapy. While this could technically influence the genetic landscape of the sequenced tumor samples, we did not observe any correlation between samples with prior exposure and higher mutational burdens. Cohort 2 additionally was derived from a study looking into the efficacy of ixabepilone ± bevacizumab treatment on chemo-resistant tumors. The study was not exclusive to any one ovarian tumor type and thus cohort 2 contains multiple histologies, two different treatment regimens, and sample DNA extracted from FFPE tissues, requiring additional cleanup steps during the analysis. And unlike cohort 1, cohort 2 did not have access to matched normal samples for CNV calling and was instead called against a process-matched panel of normals. The small cohort sizes and other confounding factors mentioned here also limited our achievable statistical significance. Thus, we were only able to report nominally significant results at the individual gene level. Lastly, ovarian cancer is a complex, heterogeneous disease that can have different genetic alterations in different parts of the tumor, as showcased by recent publications using spatial transcriptomics [37,38]. This would make follow-up studies using large cohort sizes with integrated multi-omics approaches and available independent validation datasets an exciting next step for the future to further investigate or explore findings.

## 4. Materials and Methods

### 4.1. Patient Population

Fresh tumor samples from a group of surgically resected, mostly chemotherapy-naïve patients (cohort 1) [6], and FFPE archival tissue from platinum-resistant/refractory epithelial ovarian cancer samples (cohort 2) [8,9] were collected from 49 (cohort 1) and 31 (cohort 2) advanced stage ovarian cancer patients. All but two tissue samples for DNA extraction were obtained from primary tumors that had undergone at most 2–6 cycles of neoadjuvant chemotherapy. The two remaining cases (marked as multiple prior treatments in Table 2) were obtained from tissue collected after multiple rounds of various chemotherapies. Blood samples or normal (non-tumoral) tissues were available for all patients included in cohort 1 to perform matched tumor/normal tissue analysis and eliminate germline variants during bioinformatics evaluation of somatic variants. All patients included in the study were either diagnosed and treated at Yale University School of Medicine, New Haven, CT or at the University of Brescia School of Medicine, Brescia, Italy. Cohort 2 tumor samples were retrieved from patients enrolled within a randomized phase II trial of weekly ixabepilone ± biweekly bevacizumab for platinum-resistant or refractory ovarian/fallopian tube/primary peritoneal cancer at Yale University School of Medicine (NCT03093155) [8,9]. The clinical data, including age, date of diagnosis, FIGO stage, the histological type and grade of the tumor, residual disease after surgery, oncological treatment and resistance status for cohorts 1 and 2 were obtained from medical records. The resistance status was defined according to Markman and Hoskins criteria [3] that is based on the platinum-free interval (PFI) measured as the time from cessation of the platinum-based chemotherapy to disease recurrence or progression. Patients with PFI < 6 months were considered platinum-resistant, and patients with PFI ≥ 6 months were platinum-sensitive. Patients progressing during the CP regimen were considered platinum refractory. In cohort 1, 12% (6/49) of patients received neoadjuvant chemotherapy (2–6 cycles of carboplatin + paclitaxel) before the collection of the tumor. In cohort 2, 42% (13/31) of patients had prior exposure to chemotherapy before staging surgery, with most of those (11/13) being neoadjuvant. All patients completed first-line chemotherapy with regimens based on a combination of platinum (carboplatin or cisplatin) and taxane paclitaxel. One patient from the sensitive group received platinum monotherapy. All patients included in the study signed informed consent to allow the collection of the samples. This retrospective study followed the ethical standards and agreed with the 1964 Helsinki Declaration and its later amendments or comparable ethical standards. The Yale Institutional Review Board also approved the study protocol.

### 4.2. Whole-Exome Sequencing

Genomic DNA from a total of 80 ovarian cancer samples and 49 matched normal tissues was used for WES analysis. Samples were sequenced on Illumina NovaSeq 6000 instruments (San Diego, CA, USA) using Roche Exome V2 (Indianapolis, IN, USA) and IDT xGen Exome V2 (Coralville, Iowa, USA) kits. Tumor samples were sequenced at about 200 million reads with mean +/− SEM 20x coverage of 94.1% +/− 0.3% and matched normal samples at about 100 million reads and mean +/− SEM 20x coverage of 90.0% +/− 0.3%. Sequencing data were analyzed as previously described [39]. Briefly, analysis was performed following the GATK Best Practice workflow [40,41,42,43,44,45] using standard parameters (GATK version 4.4.0.0) and a panel of normals generated separately from prior samples sequenced using similar modalities. FFPE filtering was performed with FFPolish (v0.1) [46] using default parameters. Somatic copy number variation analysis was performed using Facets (v0.5.14) [47,48], Sequenza (v3.0.0) [49] and CNVkit (v0.9.12) [50,51,52] for samples with matching normal with a median threshold between the callers of CN ≥ 4 considered as amplification, and using PureCN (v2.12.0) [53,54], Sequenza and CNVkit for samples without matching normal using a process-matched panel of normals and a median threshold between the callers of CN ≥ 5 considered as amplification. GISTIC2.0 [55,56,57] was used for enrichment analysis using default parameters.

### 4.3. Analysis of HRD Mutational Signatures

WES data from ovarian cancer specimens with DNA extracted from fresh tumor tissues with matched normal DNA were analyzed for mutational signatures of HRD using multiple modalities including the use of the software ovaHRDscar (v0.1.0) [58], which analyzes the samples’ allelic imbalance by quantifying LOH events (considering events between 10 and 50 Mb), large-scale transitions (which considers two events of at least 12 Mb that are no more than 1 Mb apart), and telomeric allelic imbalances (events that reach up to the telomere but not beyond the centromere), and then combines the individual scores into an overall score by adding them up. We used a threshold of final score >= 42 as originally proposed by Telli et al. [59] to classify samples as HRD. This threshold provided the right amount of sensitivity for our data to accurately make HRD calls while avoiding sensitivity issues leading to false-negative calls. In addition, we validated the obtained calls by analyzing somatic mutations across three mutation channels, single base substitutions (SBS), small insertions and deletions, and copy number alterations, across all 49 serous ovarian cancer exomes. Mutational matrices were constructed using SigProfilerMatrixGenerator (v1.3.6) [60] with GRCh37 as the reference genome. Extraction was performed independently for each mutation type using SigProfilerExtractor (v1.2.7) (v1.2.6) [61] with the exome flag enabled. De novo signatures were decomposed into COSMIC reference signatures (v3.5), yielding SBS1, SBS5, and SBS3 for SBS; ID6, ID8, and ID9 for indels; and CN1, CN2, CN9, CN13, CN17, and CN22 for copy number alterations. A multi-modal signature-based HRD classification was applied using the decomposed signature activity counts. Samples with detectable activity (≥ 1 mutation attributed) in any of the HRD-associated signatures, specifically SBS3 in the SBS channel, ID6 or ID8 in the ID channel, and CN17 in the CN channel, were considered positive in each respective channel. These signatures have been previously established as hallmarks of homologous recombination deficiency [62]. Samples were classified as high-confidence HRD if HRD-associated activity was detected in two or more channels, low confidence if in exactly one channel, and no evidence if none. Orthogonally, HRD status was independently assessed using HRProfiler (v0.0.2) [63], a support vector machine model that uses six mutational patterns derived from WES data to predict HRD status. The ovarian WES model was applied to all 49 samples, and samples with an HRD probability ≥ 0.6 were classified as HRD. The probability threshold of 0.6 is slightly more conservative than the 0.5 threshold used in the original publication [63] and was chosen to minimize false positive calls. Consensus HRD status was defined as HRD if both approaches independently classified a sample as HRD, HRP if both classified a sample as HRP, and discordant otherwise.

### 4.4. Statistical Analysis

The Pearson’s chi-square test (for factorial comparisons) or the Mann–Whitney test (factorial versus continuous data) were used to analyze differences in mutation load, mutational signatures, and CNVs between patients, stratified by clinical data. Survival functions for groups of patients divided by genetic and clinical data were plotted using the Kaplan–Meier method, and significance was calculated by log-rank test. Effect size estimates were reported as hazard ratios with corresponding *p*-values and 95% confidence intervals using a Cox regression model. Histology and treatment arm were included as covariates and fitting was performed using the Python (v3.11.6) lifelines implementation. A two-sided *p*-value of less than 0.05 was considered significant. *p*-values were corrected for multiple testing using the Benjamini–Hochberg false discovery rate (FDR) procedure. An adjusted *p*-value < 0.05 was considered statistically significant and if not reached, *p*-values < 0.05 were reported as nominally significant. The Graphpad Prism (v10.6.1) program and Scipy (v1.11.3) python package were used for statistical analyses. CbioPortal was used to further validate gene specific copy numbers and overall survival for the OV-TCGA cohort selecting high-grade serous cases and comparing putative copy numbers while classifying either Gain or Amp as equivalent to amplification and any loss as equivalent to loss.

## Figures and Tables

**Table 1:** Clinical characteristics of ovarian cancer patients included in cohort 1.

Patient	Age at Diagnosis	FIGO Stage	Histology	Race	Prior Treatment
OVAP_10	66	IIIC	HGSC	White	-
OVAP_11	78	IIIC	HGSC	White	-
OVAP_12	50	IIIC	HGSC	White	-
OVAP_13	58	IIIC	HGSC	White	-
OVAP_15	61	IIIB	HGSC	White	-
OVAP_18	57	IIIC	HGSC	White	-
OVAP_1	53	IVB	HGSC	White	-
OVAP_20	60	IVA	HGSC	White	-
OVAP_22	69	IVB	HGSC	White	-
OVAP_23	38	IIIC	HGSC	White	-
OVAP_24	80	IIIC	HGSC	White	-
OVAP_25	58	IVA	HGSC	White	-
OVAP_26	46	IVB	HGSC	White	-
OVAP_27	68	IIIC	HGSC	White	-
OVAP_28	63	IIIC	HGSC	White	-
OVAP_29	44	IIIC	HGSC	White	-
OVAP_2	67	IIIC	HGSC	White	-
OVAP_30	50	IIIC	HGSC	White	-
OVAP_32	24	IVB	HGSC	White	-
OVAP_33	60	IIIC	HGSC	White	-
OVAP_36	45	IIIC	HGSC	White	-
OVAP_38	57	IIIC	HGSC	White	-
OVAP_3	61	IIIC	HGSC	White	-
OVAP_42	41	IIC	HGSC	White	-
OVAP_45	62	IIIC	LGSC	White	-
OVAP_46	74	IIIC	HGSC	White	-
OVAP_47	56	IIIC	LGSC	White	-
OVAP_48	50	IIIC	HGSC	White	-
OVAP_49	66	III	HGSC	White	Neo
OVAP_4	64	IIIC	HGSC	White	-
OVAP_51	48	IIIC	HGSC	White	-
OVAP_52	42	IIIC	HGSC	White	Neo
OVAP_54	66	IVB	HGSC	White	-
OVAP_55	54	IIIC	HGSC	White	-
OVAP_57	73	IIIC	HGSC	White	-
OVAP_58	54	IIB	HGSC	White	-
OVAP_59	69	IIIC	HGSC	White	-
OVAP_60	71	IIIC	HGSC	White	Neo
OVAP_6	50	IIIC	HGSC	White	-
OVAP_7	65	IVA	HGSC	White	-
OVAP_9	52	IIIC	HGSC	White	-
OVAP1	53	IVB	HGSC	White	Neo
OVAP2	66	IIIC	HGSC	White	-
OVAP26	60	IIIC	HGSC	White	Neo
OVAP27	68	II	HGSC	White	-
OVAP29	44	IIIC	HGSC	White	Neo
OVAP3	55	IIIC	HGSC	White	-
OVAP6	33	IIIC	HGSC	White	-
OVAP7	55	IIIC	HGSC	White	-

FIGO: International Federation of Obstetrics and Gynecology; HGSC: high-grade ovarian serous carcinoma. LGSC: low-grade ovarian serous carcinoma. Neo: neoadjuvant treatment.

**Table 2:** Clinical characteristics of ovarian cancer patients included in cohort 2.

Patient	Age at Diagnosis	FIGO Stage	Histology	Race	Arm	Prior Treatment
IXA49	76	IIIC	HGSC	African-American	B	-
IXAY01	57	IIIC	HGSC	White	B	-
IXAY021	67	IVB	HGSC	White	A	-
IXAY023	69	IIIC	HGSC	White	B	-
IXAY024	68	IIIC	HGSC	White	A	-
IXAY029	54	IIIC	HGSC	White	B	Neo
IXAY03	59	IIIC	HGSC	White	B	Neo
IXAY047	65	IIIC	HGSC	White	A	Neo
IXAY05	39	IVB	HGSC	White	B	-
IXAY10	43	IIIC	HGSC	African-American	A	Multi
IXAY19	67	IIIC	Carcinosarcoma	White	B	-
IXAY22	64	IIIC	HGSC	White	B	-
IXAY25	62	IIIC	HGSC	White	B	Neo
IXAY31	69	IIIC	HGSC	White	B	-
IXAY34	56	IIIC	HGSC	White	B	-
IXAY37	76	IIIC	HGSC	White	B	Neo
IXAY38	73	IIIB	HGSC	Hispanic	B	-
IXAY48	55	IC	HGSC	White	B	-
IXAY51	54	IVA	HGSC	African-American	B	Neo
IXAY53	71	IIIC	HGSC	White	B	-
IXAY57	69	IVB	HGSC	African-American	A	-
IXAY58	59	IIIC	Clear-cell	African-American	A	Multi
IXAY59	74	IIIC	Clear-cell	White	B	-
IXAY60	54	IVB	HGSC	White	B	-
IXAY61	61	IVB	HGSC	White	A	Neo
IXAY64	68	IIIC	HGSC	White	B	Neo
IXAY65	61	IVA	HGSC	African-American	A	Neo
IXAY66	56	IIIC	HGSC	African-American	B	Neo
IXAY69	73	IVB	Carcinosarcoma	White	A	Neo
IXAY70	67	IIIC	HGSC	White	B	-
IXAY73	70	IVB	HGSC	White	A	-

FIGO: International Federation of Obstetrics and Gynecology; HGSC: high-grade ovarian serous carcinoma. Neo: neoadjuvant treatment. Multi: multiple prior treatments.

**Table 3:** Gene characteristics for genes amplified in CP-resistant patients in cohort 1.

Pathway	Member Genes
PI3K/AKT signaling	*ATG5*, *EPHA7*, *ETNK1*, *FOXO3*, *FYN*, *MAP3K7*, *PIK3C2G*, *ROS1*, *SESN1*, *SGK1*
Immune/Inflammatory signaling	*BACH2*, *FOXO3*, *IFNGR1*, *MAP3K7*, *NOD1*, *PRDM1*, *SGK1*, *TNFAIP3*
TGF-beta signaling	*ACVR2A*, *FOXO3*, *INHBA*, *RSPO3*, *RUNX2*, *SFRP4*
MEK/ERK signaling	*EPHA7*, *ETV1*, *FYN*, *MAP3K7*, *MYB*, *PTPRK*, *PTPRO*, *ROBO1*, *ROS1*

**Table 4:** Gene characteristics for genes correlated to longer overall survival in cohort 2.

Relationship	Member Genes
Chemo-resistance-related	*AKT3*, *BCL9*, *CKS1B*, *CLPTM1L*, *JAG1*, *MCL1*, *MUC1*, *PARP1*, *PCNA*, *PRKAA1*, *RAB25*, *RICTOR*, *TERT*, *TRIB3*, *TRIP13*
Microtubule-related	*CAMSAP2*, *CENPA*, *CEP72*, *DPYSL5*, *FMN2*, *KIF14*, *KIF16B*, *KIF21B*, *KIF26B*, *KIF3C*, *MAPRE3*, *NES*, *TBCE*, *TPPP*, *TRIM46*
Cytoskeleton/structural scaffold-related	*ACTA1*, *ACTN2*, *DSTN*, *LMNA*, *SPTA1*, *TPM3*

## Data Availability

Data will be made available upon reasonable request.

## Abbreviations

The following abbreviations are used in this manuscript:

CP	carboplatin/paclitaxel
OC	ovarian cancer
IB	ixabepilone ± bevacizumab
WES	whole exome sequencing
CR/PR	complete/partial response
SD/PD	stable/progressive disease
PFS	progression-free survival
HGSOC	high-grade serous ovarian cancer
CNV	copy number variant
LOH	loss of heterozygosity
PFI	platinum-free interval
OS	overall survival
